# Sediment delivery of partially-unfrozen loam soil rill by snow/glacier meltwater flow

**DOI:** 10.1038/s41598-019-40400-4

**Published:** 2019-03-08

**Authors:** Dan Wan, Fa-hu Li, Wu Yu, Chao Chen, Yuan Gao

**Affiliations:** 1 College of Water Resources and Civil Engineering, China Agricultural University, Beijing, 100083 China; 2 Department of Resources and Environment, Tibet Agricultural and Animal Husbandry College, Linzhi, 860000 China

## Abstract

Erosion of freeze-thaw soil by meltwater from snow/glacier is one of the main erosion types in high altitude or latitude regions. This study aims to experimentally measure soil erosion processes over partially-unfrozen soil slopes in laboratory. The experiments including three slope gradients of 10°, 15°, and 20°, three water flow rates of 1, 2, and 4 L/min (0.06, 0.12, and 0.24 m^3^/h), and three thawed-soil depths of 1, 2, and 5 cm were conducted to measure sediment concentration and calculate its delivery rate under seven slope lengths of 0.5, 1.0, 2.0, 3.0, 4.0, 5.0, and 6.0 m. The sediment delivery rates from nonfrozen soil slopes under the corresponding slope gradients, flow rates, and slope lengths also were measured as control treatments. Results showed that the sediment delivery rate from both partially-unfrozen and nonfrozen soil slopes increased logarithmically with slope length. The sediment delivery rate from partially-unfrozen soil slope increased with the increased slope gradient and meltwater flow rate significantly, and the effect of water flow rate on it was greater than that of slope gradient. The thawed-soil depth did not significantly affect sediment delivery rate. The sediment delivery rate from a partially-unfrozen loamy soil slope averagely was 11.4% smaller than that from nonfrozen soil slope. This study is helpful to understand the erosion process of thawing-soil by meltwater from snow/glacier.

## Introduction

Freeze-thaw (FT) soil erosion is one of the soil erosion types in high altitude and/or high latitude regions^1–4^, and the area suffering FT erosion is about 1.28 million km^2^ in China and accounts for 13.31% of its total arable land acreage^5^. The soil loss process under FT condition is one of main concerns because its water cycle process and soil erosion mechanism are obviously different from nonfrozen soil. Especially for the cultivated slope land without protective covers by vegetation, overland flow is easily concentrated and hence dramatically increases its kinetic energy and subsequently soil loss.

The FT process destroys soil physical properties such as aggregate stability and soil structure^6–9^, changes soil density and soil strength^10,11^, and hence results in the increase of soil erodibility and deteriorates soil loss^12^. Scientists have paid great attention on FT soil erosion. The influences of climate, terrain, or land use type on it^13,14^, as well as the meltwater runoff process and its seasonal variation^14–16^ have been studied intensively. The soil erosion under FT condition is affected by temperature, soil water content, rainfall intensity or meltwater flow rate and others, and the greatest environmental factor for meltwater erosion is rainfall intensity or water flow rate^17^.

Soil FT cycle affects soil erodibility. The result of indoor simulated rainfall experiment conducted by Edwards and Burney showed that the soil loss on bare soil slope after being frozen and thawed increased by 90%^18^, and the FT process resulted in about 90% and 25% increase of sediment mass in interrill and rill, respectively^19^. A more serious soil erosion was observed in spring than others because of its higher soil erodibility during this period^17,20–23^. The soil erosion amount from a partially-unfrozen soil slope could increased by 1.4–4.0 times than that from a nonfrozen soil slope, and this multiple would increase with the increase of initial soil water content^21^.

Moreover, a partially-unfrozen soil also changes water circle process. Under the partially-unfrozen soil, there exists an impermeable layer below the thawed soil that prevents water infiltrates into deeper soil layer^24^, and hence results in an increased lateral subsurface flow and a serious soil erosion loss in spring^25,26^. Moreover, a low friction on the interface between the thawed soil layer and its beneath frozen soil layer results in a greater overland runoff velocity^27–29^. The thawing of soil also increases its water content. These also are responsible to the high runoff, great sediment content, and serious soil erosion loss in spring^30,31^.

An enormous studies have been conducted on the effect of FT cycle on soil erosion, however the studies on the process and mechanism of soil erosion caused by meltwater in high altitude cold regions are rare due to the limitation of field observation and monitoring technology. Moreover, the research of thawed-soil depth impact on sediment yield under different hydrodynamic conditions is less reported, and this is important to understand the mechanism of soil erosion during thawing process and is the basic to simulate soil erosion process under FT condition. Sediment delivery rate represents the net result of multiple processes from sediment detachment to deposition through transport, and it comprehensively describes the intensity of soil rill erosion. Thus, this study aims to (1) study sediment delivery during the soil thawing process; (2) determine the variation of sediment delivery rate on partially-unfrozen soil with slope length, slope gradient, thawed depth, and meltwater rate; and (3) analyse their impacts on sediment delivery rate of partially-unfrozen soil slope.

## Results

### Sediment delivery rate

**Figure 1:**
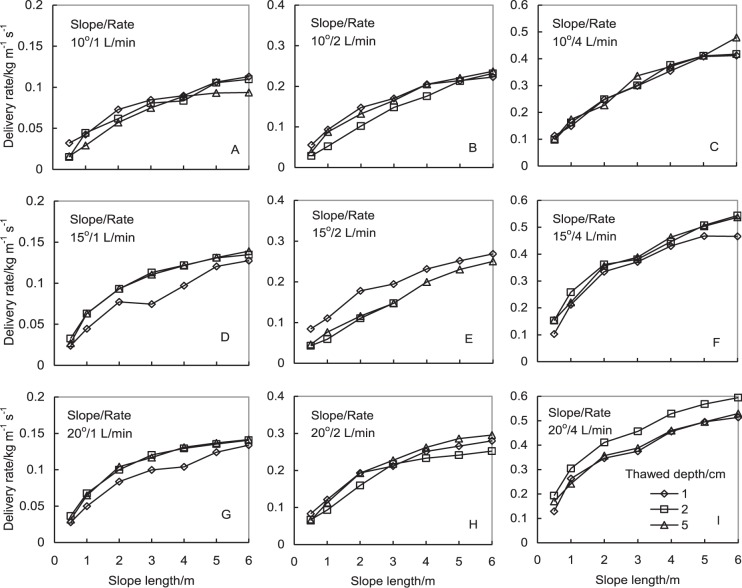
Variation of sediment delivery rate along with slope length at the slope gradients of 10° (above row), 15° (middle row), and 20° (below row) as well as the meltwater flow rates of 1 (left column), 2 (middle column), and 4 L/min (right column) under different thawed depths of 1, 2, and 5 cm.

The variation of sediment delivery rate with slope length is shown in Fig. 1 under different thawed-soil depths, slope gradients, and meltwater flow rates. The standard deviations were not shown in the figure for the sake of clarity. The sediment delivery rate from partially-unfrozen slopes generally increased with slope length logarithmically under the different thawed depths and flow rates (*R*^2^ ≥ 0.973^***^), and their increased rate (the gradient of the curve in Fig. 1) decreased gradually with the increased slope length. The sediment delivery rate from nonfrozen soil slope also showed a similar variation tendency to partially-unfrozen slope with slope length (*R*^2^ ≥ 0.987^***^, data not shown). The effect of thawed depth on sediment delivery rate was not significant (Fig. 1).

Water flow rate significantly increased sediment delivery rate, and a greater water flow rate resulted in a greater sediment delivery rate (Fig. 1), which is related with runoff kinetic energy. Slope gradient also increased sediment delivery rate, but its effect on it was relative small as compared with water flow rate (Fig. 1). When flow rate was 1 L/min, the averaged sediment delivery rate on 6 m long slope was 0.105 ± 0.010, 0.134 ± 0.006, and 0.139 ± 0.004 kg/(m s) respectively for the slope gradients of 10°, 15°, and 20° under three tested thawed depths. The corresponding values were 0.436 ± 0.037, 0.516 ± 0.043, and 0.546 ± 0.043 kg/(m s) when the flow rate was 4 L/min.

### Impacts of tested factors on sediment delivery rate

A partial correlation analysis was made to analyse the influence degree of different tested factors on sediment delivery rate of partially-unfrozen soil slopes and analysis results are shown in Table 1. The results indicated that sediment delivery rate had a significantly positive correlation with slope gradient, water flow rate, and slope length at the 0.001 probability level. The correlation coefficient of sediment delivery rate with water flow rate was the greatest, that with slope length was the second, and the slope gradient was the smallest. The effect of thawed depth on sediment delivery rate was not statistically significant.

**Table 1:** Partial correlation coefficient among sediment delivery rate and tested experimental factors.

	Slope gradient	Flow rate	Thawed depth	Slope length
Sediment delivery rate	0.451***	0.916***	0.028	0.838***

Note: ***Indicates significant at the 0.001 probability level (2-tailed).

### Comparison of sediment delivery rates between partially-unfrozen and nonfrozen soil slopes

**Figure 2:**
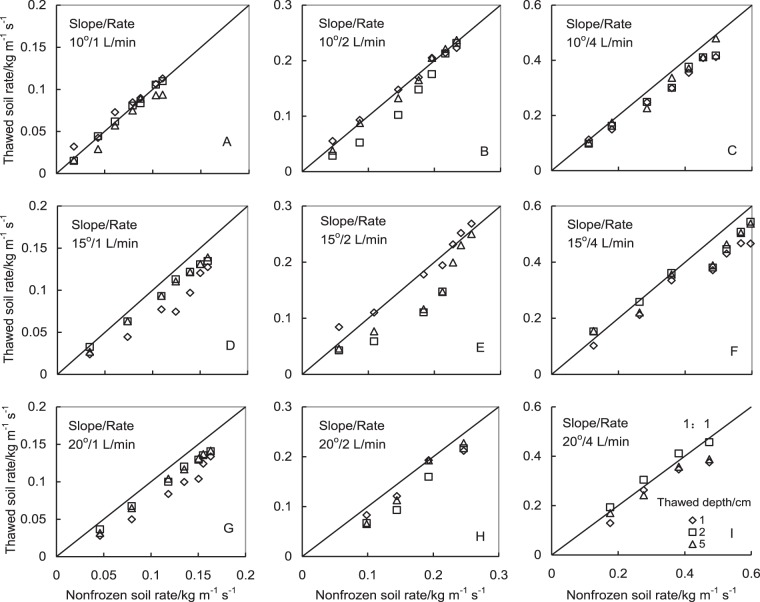
Comparison of sediment delivery rates between partially-unfrozen and nonfrozen loamy soil slopes at the slope gradients of 10° (above row), 15° (middle row), and 20° (below row) as well as the meltwater flow rates of 1 (left column), 2 (middle column), and 4 L/min (right column) under different thawed depths of 1, 2, and 5 cm.

As shown in Fig. 2, the FT process exerted an obvious effect on soil rill erosion rate, and the sediment delivery rate from partially-unfrozen soil slope generally was smaller than that from nonfrozen soil slope except for a few treatments.

In order to distinguish the influence of FT behaviour on rill erosion further, the ratio (*K* value) of the sediment delivery rate from partially-unfrozen loamy soil slope to that from nonfrozen soil slope is calculated and shown in Table 2. A linear relationship between sediment delivery rates from partially-unfrozen and nonfrozen soil slopes existed with a significant correlation coefficient under different slope gradients and flow rates (Table 2). These data in Table 2 indicates that the sediment delivery rate from partially-unfrozen loamy soil slope was positively proportional to that from nonfrozen slope. The averaged *K* value of 0.886 ± 0.092 for all experimental treatments confirms that the sediment delivery rate from partially-unfrozen soil slope generally was smaller than that from nonfrozen slope by about 11.4% averagely.

**Table 2:** Correlation relationships between the sediment delivery rate from partially-unfrozen soil slope and that from nonfrozen soil slope.

Slope gradient (°)	Flow rate (L/min)	Thawed depth (cm)	*K*	*R* ^2^
10	1	1	1.052	0.994***
		2	1.001	0.999***
		5	0.907	0.993***
	2	1	0.995	0.998***
		2	0.899	0.985***
		5	0.997	0.998***
	4	1	0.861	0.999***
		2	0.878	0.999***
		5	0.920	0.997***
15	1	1	0.728	0.988***
		2	0.870	0.999***
		5	0.872	1.0***
	2	1	1.014	0.995***
		2	0.645	0.991***
		5	0.849	0.976***
	4	1	0.813	0.997***
		2	0.892	0.994***
		5	0.888	0.995***
20	1	1	0.748	0.993***
		2	0.864	1.0***
		5	0.866	0.999***
	2	1	0.896	0.994***
		2	0.812	0.988***
		5	0.903	0.988***
	4	1	0.849	0.993***
		2	1.029	0.996***
		5	0.873	0.996***

Note: *K* is the ratio of sediment delivery rates between partially-unfrozen soil slope and nonfrozen soil slope; ***Indicates significant at the 0.001 probability level.

Under the gentle slope gradient of 10°, the *K* value decreased with the thawed depth as flow rate was 1 L/min, but it was on the contrary as the flow rate was 4 L/min (Table 2). However, when slope gradient was greater than 15°, the *K* value increased to a steady or a maximum value with the increase of thawed depth at the both flow rates of 1 and 4 L/min, which possibly indicates the insufficient sediment supply from partially-unfrozen loamy soil slopes. Under the water flow rate of 2 L/min, the variation tendency of *K* values under all tested 3 slopes firstly decreased and then increased with the increase of thawed depth, which possibly is the influence result of subsurface flow in soil on runoff proportion^17^. At the thawed depth of 2 cm and the water flow rate of 2 L/min, the smaller *K* value indicates a relatively less soil detachment but a relatively greater runoff amount from partially-unfrozen soil and its reason is not clear because only sediment concentration in runoff was measured in the study.

## Discussion

Soil erosion involves the granular particle detachment from soil aggregate by runoff, particle transport with runoff, and particle settlement from runoff, all these processes occur simultaneously and they maintain a dynamic equilibrium. When water flows on a soil slope, it scours soil bed and detaches soil aggregate. Soil particles are suspended in runoff if runoff kinetic energy is enough to carry them, and on the contrary, they will settle down to deposit. Sediment delivery rate represents a net result between soil particle detachment and its settlement from runoff, and it depends on soil detachment capability and sediment carrying capacity by runoff, which is determined by soil property and runoff kinetic energy together.

### Effects of slope length, slope gradient, and flow rate

Similar to normal nonfrozen soil slope, runoff from a partially-unfrozen soil slope carries the detached particles and moves them toward the slope outlet. Water flow keeps detach soil particles along the rill and subsequently its sediment delivery rate increases with slope length because more soil particles enter into runoff. The increased sediment load in runoff decreases the increased rate of sediment delivery rate due to the settlement of some suspended matters, which results in that the sediment delivery rate logarithmically increased with the increased slope length (Fig. 1).

The increased water flow rate and slope gradient increase runoff kinetic energy, which necessarily increases the sediment delivery rate on partially-unfrozen soil slope significantly if the soil erodibility is not a limiting factor (Fig. 1). The partial correlation analysis result shown in Table 1 demonstrates that the influence of water flow rate on sediment delivery rate was the greatest, the slope length the second, and the slope gradient the third in all three tested factors involving slope parameters under experimental conditions. The result may provide a hint on the selection of soil conservation engineering measures. Shortening the length of soil slope by contour hedgerow establishment or engineering measure maybe is more effective, cheaper, and more practical means to prevent soil loss by erosion than the variation of slope gradient in the regions suffering FT actions if meltwater runoff cannot be controlled economically.

### Effect of thawed-soil depth

Sediment delivery rate involves the equilibrium between the source and sink of soil particles suspended in runoff, and the variations of soil erodibility and the soil detachment capacity affected by surface runoff may affect its value. When the thawed-soil depth increases, sediment supply increases and more soil particles can be detached and enters into runoff. Therefore, the sediment delivery rate should increase with the increase of thawed depth. However, the increase of thawed-soil depth also increases the possibility of more subsurface flow in soil, and hence decreases the water amount on soil surface and the energy to detach soil. Both reasons possibly resulted in that the effect of thawed depth on sediment delivery rate was not significant (Fig. 1 and Table 2). Moreover, the thawed-soil depth was determined by a probe method in the study, operators’ influence is great, and this also can cause some deviation on experimental results.

### Effect of thawing process

Unlike the studies on the effect of freeze-thaw cycle reported in most articles, this study focused on the effect of thawing process on soil erosion. The FT cycle of soil deteriorates its aggregates stability and changes soil hydraulic properties, and consequently increases soil loss^7,9,19^. However, the properties of a thawing soil are different from those of nonfrozen soil or the soil rebalanced with its surrounding environment after FT cycle. There exist some ice fragments in the thawing soil layer and a hard frozen soil layer below the shallow thawed-soil layer during thawing process. Our experimental result demonstrated that the sediment delivery rate from a partially-unfrozen soil slope was generally smaller than that from a nonfrozen slope except the few treatments (Fig. 2 and Table 2). Maybe it is due to nonsufficient soil particle supply caused by the shallow thawed-soil depth. Moreover, the soil under thawing condition possibly is not thawed completely, some ice fragments exist and its temperature is lower than 0 °C, and hence increases water viscosity and decreases the detachment capacity of partially-unfrozen soil as compared with nonfrozen soil. The exact reason that resulted in the smaller sediment delivery rate under partially-unfrozen soil slope is not known and it needs to be studied further.

## Conclusions

The influence mechanism on sediment delivery from a partially-unfrozen soil slope is complicated. The study results showed that the variation of sediment delivery rate increased logrithmatically with the increase of slope length over partially-unfrozen and nonfrozen soil slopes. The water flow rate and slope gradient imposed significant effects on sediment delivery rate from a partially-unfrozen slope, and the influence of water flow rate was greater than that of slope gradient. However, the effect of thawed-soil depth on it was not significant. The sediment delivery rate from a partially-unfrozen soil slope generally was smaller than that from nonfrozen soil slope, and it was about 88.6% of the latter averagely. The study is helpful to further understand the erosion mechanism of partially-unfrozen soil.

## Methods and Materials

### Test apparatus and experimental procedures

The tested soil was collected from 0–30 cm surface layer of a mountain farmland (N39°46′, E115°35′, 850 m a.s.l.) in Fangshan District, Beijing. It locates at the junction zone of North China Plain and Taihang Mountain in the southwest of Beijing Municipality with a month-averaged temperature of −5.0–24.4 °C and a precipitation of 645.2 mm, which suffers an obvious FT action. The collected soil was silt loam soil with the sand of 34.8% (0.05–2 mm), the silt of 50.2% (0.002–0.05 mm), and the clay of 15.0% (<0.002 mm).

The experimental system was set up with a steel flume and a plastic water-supply tank^32^. For convenient movement between the freezing chamber and the test platform in laboratory, each flume section was designed to be 3.0 m long, 0.1 m wide, and 0.12 m deep. Two sections were jointed together to form an experimental soil flume with 6.0 m in length, with a steel plate of 0.1 m high at the end of the soil flume. Different slope lengths could be obtained by adjusting the inlet position of water flow along the 6.0 m-long soil flume, and the inclination angle of test platform can be adjusted in the range of 0°–35° according to the experimental design^4^.

The collected soil was air-dried and passed through a 10 mm sieve to remove stone and grass roots. The prepared soil materials were filled into soil flume to a depth of 10 cm, with no compaction. After the soil flume was filled with the tested soil, it was laid on the horizontal status, and the soil in the flume was slowly saturated for 24 h to assure the homogeneity of soil moisture content and get an even soil bulk density. After that, the soil flumes were kept in a freezing chamber for more than 24 h under a temperature range of from -25–-15 °C. After the soil was completely frozen, the flumes were taken out of the freezing chamber and laid on the test platform to thaw at the room temperature in laboratory.

The thawing system of frozen soil was composed of side ice trough, bottom ice trough, and insulation materials. The side and the bottom ice troughs were affiliated on the soil flume. In order to assure the one-dimensional thawing of the frozen soil from soil surface, the soil flume was put on the bottom ice trough directly. The gap between the side of soil flume and the side ice trough was filled with heat-insulating sponge to avoid heat loss from the horizontal and bottom directions of soil flume. These test measures help to realize one-dimensional vertical thawing of the frozen soil down from its surface layer^27,33^. Based on probe test method, the thawing duration to reach a designed thawed-soil depth can be determined.

The experiments were started after the soil in the flume was thawed to the designed depth. In order to simulate natural meltwater from snow/glacier and avoid the influence of heat conduction and exchange, a 200 L water tank that was filled with the mixture of water and ice was employed in the experiments, and the temperature of water from the tank was near to 0 °C. After all preparation about experiment has been completed, the test platform was adjusted to the design slope gradient. The experimental water was supplied into a steady current groove, which was 0.5 m long, from the water tank by a peristaltic pump, and then flowed into the upper end of the soil flume. Water supply rate range was 0–60 L/min and it can be adjusted according to experimental design requirement.

Four sediment-laden water samples were taken continually to measure their sediment concentrations after runoff status was steady for different designed flow rates, slope gradients, and slope lengths. The time that runoff got to be steady was about 30 min. The samples were taken manually with small cups of approximately 150 cm^3^ in volume, at the end of the flume. The collected samples were weighed and oven-dried at 105 °C oven for more than 8 h before they were weighed again to determine their sediment concentrations.

When an experiment for a given slope length was finished, the position that water flow entered the soil flume was adjusted by moving the steady flow trough forward, and another slope length could be tested.

### Experimental treatments

The experimental treatments included three slope gradients of 10°, 15°, and 20°, three water flow rates of 1, 2, and 4 L/min (0.06, 0.12, and 0.24 m^3^/h), three thawed-soil depths of 1, 2, and 5 cm, and seven slope lengths of 0.5, 1.0, 2.0, 3.0, 4.0, 5.0, and 6.0 m. A series of experiments with the corresponding slope gradients, flow rates, and slope lengths for nonfrozen soil also were conducted as control treatments, and their experimental procedures were the same as that did for the partially-unfrozen soil slope except for freeze-thaw process. All treatments in the study were replicated triplicate.

### Sediment delivery rate calculation

Sediment concentration was calculated by the following formula:

1 $$C=\frac{{m}_{s}}{V}$$

where *C* is sediment concentration, kg/m^3^; *V* is the volume of runoff sample, m^3^; *m*_*s*_ is the sediment mass in runoff sample, kg.

Sediment delivery rate was calculated according to Eq. 2:

2 $$T=Cq$$

where *T* is sediment delivery rate, kg/(m s); *C* is sediment concentration, kg/m^3^; *q* is the water flow rate per unit width, m^2^/s.

### Statistical analysis

The significance of differences among treatment means was tested by one-way ANOVA, using SPSS 21.0 (SPSS Inc., Chicago). When the *F* value in the ANOVA was statistically significant, a least significant difference test (*P* = 0.05) was used for separation of the means. Partial correlation analysis with a two-tailed method was applied to analyze the relationships between sediment delivery rate and different tested experimental factors.
